# Phosphoantigen Presentation to TCR γδ Cells, a Conundrum Getting Less Gray Zones

**DOI:** 10.3389/fimmu.2014.00679

**Published:** 2015-01-15

**Authors:** Gennaro De Libero, Sze-Yi Lau, Lucia Mori

**Affiliations:** ^1^Singapore Immunology Network, Agency for Science, Technology and Research (A*STAR), Singapore, Singapore; ^2^Department of Biomedicine, University of Basel, Basel, Switzerland

**Keywords:** antigen presentation, γδ TCR, butyrophilin 3A1, infection control, tumor surveillance

## Abstract

The mechanistic requirements of antigen recognition by T cells expressing a γδ TCR has revealed important differences with those of αβ TCR cells and, despite impressive new data generated in the very recent years, they remain poorly understood. Based on the structure of the TCR chains and the tissue distribution, γδ cells are represented in a variety of populations. The major subset of human peripheral blood γδ cells express Vγ9Vδ2 TCR heterodimers and are all stimulated by phosphorylated metabolites (commonly called phosphoantigens). Phosphoantigens are molecules with a very small mass and only stimulate Vγ9Vδ2 cells in the presence of antigen-presenting cells, suggesting a strict requirement for dedicated antigen-presenting molecules. Recent studies have identified butyrophilin (BTN) 3A1 as the molecule necessary to stimulate Vγ9Vδ2 cells. BTN3A1 extracellular, transmembrane, and cytoplasmic domains have different functions, including cognate interaction with the Vγ9Vδ2 TCR, binding of the phosphoantigens, and interaction with cytoplasmic proteins. This review mainly discusses the known molecular mechanisms of BTN3A1-mediated antigen presentation to γδ cells and proposes a model of phosphoantigen presentation, which integrates past and recent studies.

## Introduction

Two types of TCR can be expressed in a mutually exclusive manner by T cells made up of either αβ or γδ chains. Both receptors are heterodimers, and while the TCR γ and β genes are encoded in different loci, the TCR δ genes, being nested within the TCR α locus, are subject to deletion when the TCR α genes are rearranged. T cells expressing TCR homologous to the human TCR γ and TCR δ genes have been described in many species, indicating an important function of γδ TCR cells. Despite the enormous number of studies on γδ TCR cells, a series of issues remain poorly solved. They include the evolutionary necessity for two separate populations of T lymphocytes (1), the nature of the antigens that stimulate γδ TCR cells, and the mechanisms of antigen presentation and the role of these cells in immune response. This paper will only touch upon some of these points to review in greater detail the published studies on the most abundant population of circulating human γδ cells, their antigen specificities, and the modes of presentation of these antigens.

## The Structure of the γδ TCR

Both αβ and γδ TCRs are heterodimers linked by disulfide bonds. Some rare γδ TCR, namely those using the constant γ2 chain, lack a critical cysteine and thus form non-covalently linked heterodimers. Whether this structural difference has important functional effects remains poorly investigated.

The structures of human γδ TCR have been solved and showed pairing of the γ and δ chains, resembling that of αβ TCR heterodimers (2). The complementarity-determining region 3 (CDR3) regions of both γ and δ genes form quite large bulges on the top of the receptor, suggesting a direct involvement in antigen recognition (3). A second main aspect is that the human TCR composed of the Vγ9 and Vδ2 chains is characterized by an elbow angle at the C–V junction, which is different from that of the αβ TCR of immunoglobulins (2). It was speculated that other γδ TCR composed of non-Vγ9 and non-Vδ2 chains also show similar angles, as the residues found in the V–C interface of the solved Vγ9Vδ2 TCR are conserved in most human and mouse γ and δ genes (4). Although this unique structure might have important functional implications, no study has directly addressed this aspect.

The CDR loops of the TCR γ and TCR δ chains closely resemble those of other TCR genes, though important differences are present. The CDR1 and CDR2 loops of Vγ and the CDR1 loop of Vδ are positioned in a manner similar to those of Vβ and Vα, respectively, and they are two residues longer than their αβ TCR counterparts. In addition, in the CDR2 loop of Vδ, the C″ strand pairs with the C′ strand of the inner β-sheet of the domain, whereas in the Vα chain CDR2 loop, the C″ strand pairs with the D strand of the outer β-sheet. These structural characteristics contribute to a jagged surface of the γδ TCR, which is very different from that of a αβ TCR that binds MHC–peptide complexes, implying that the surface of the antigen-presenting molecule interacting with the Vγ9Vδ2 TCR is very different from that of an MHC molecule.

Other studies have investigated the structure of different types of γδ TCR and their mode of interaction with respective ligands. The Vδ1 chain of an MICA-reactive T cell showed a surprisingly flat surface, which is not found in other TCR structures (3), implicating an interaction very different from that of αβ TCR with MHC molecules.

A TCR recognizing the MHC class I-like molecule T22 showed dominance in this interaction of the germline-encoded residues of the junctionally recombined CDR3δ, which bound to the α helices of T22 (5), a mode of antigen recognition different from that of antibodies and MHC-restricted TCR.

## The Antigens Stimulating γδ Cells

The conservation of the γ and δ genes throughout the primate lineage suggests that these distinctions probably have important functional consequences.

Since the initial discovery of the T cell population expressing the γδ TCR, it was clear that the identification of the nature of stimulatory antigens was an important step to understanding the function of γδ TCR during immune responses. A variety of antigens have been identified and their nature has been recently reviewed in Ref. (6). Here, we briefly describe the published studies reporting antigens, which stimulate TCR γδ cells and discuss them according to their nature and expression on target cells.

## Surface Molecules Stimulating γδ TCR Cells

The list of cell surface molecules that may establish cognate interactions with the γδ TCR is large and is continuously increasing (Table 1). All of the identified surface molecules stimulate a small percentage of γδ cells and raise the important question of whether these specificities are occasional or instead should be considered as an important part of the antigenic repertoire of γδ cells. MHC molecules were found to stimulate alloreactive responses of γδ cells (7–10), and mutagenesis studies on the MHC molecule indicated that the topology of γδ TCR interaction with the MHC was distinct from that of αβ T cells (11).

**Table 1:** **Antigens stimulating γδ cells**.

γδ TCR/cell type	Antigens/restriction molecules	Reference
**HUMAN**		
Vγ (several) Vδ1	MICA, MICB	(3, 22)
Vγ (several) Vδ1	CD1c, CD1d	(12 –17)
Vγ4Vδ5	EPCR	(18)
Vγ9Vδ2	F1 ATPase, Apo A-I	(23)
Vγ9Vδ2	GroEL homolog on Daudi Burkitt’s lymphoma cells	(25)
Vγ9Vδ2	Hsp60, Hsp65	(24, 26, 27)
Vγ9Vδ2	IPP, HMBPP	(43 –45)
	Tetanus toxoid	(30, 31)
	DXS2 or Rv2272 peptides	(40)
Vδ1 clones	HLA-A24, HLA-A2	(9, 10)
Vγ4Vδ1	HLA-B27	(32)
Vγ3Vδ2	Histidyl-tRNA synthetase	(39)
**MOUSE**		
Hybridoma G8	T10, T22, T27	(5, 11, 21)
Hybridoma KN6	T27	(11, 19)
Hybridoma LBK5	IE^k^	(11)
Vγ1Vδ8 (NX6)	Cy3	(41)
Vγ4Vδ4 (1G9)	NP	(41)
Vγ1–Jγ4/Vδ5 (MA2)	PE	(42)

*Apo A-I, apolipoprotein A-I; CD1c or CD1d, cluster of differentiation 1 isoforms; Cy3, cyanine 3; DXS2, mycobacterium 1-deoxy-d-xylulose-5-phosphate-synthase 2; EPCR, endothelial protein C receptor; HLA, human leukocyte antigen; HMBPP, (E)-4-hydroxy-3-methyl-but-2-enyl pyrophosphate; Hsp, heat-shock protein; IPP, isopentenyl pyrophosphate; MICA or MICB, MHC class I chain-related protein A or B; NP, 4-hydroxy-3-nitrophenylacetyl; Rv2272, mycobacterium transmembrane protein; T10, T22, or T27, MHC class Ib proteins; PE, algae protein phycoerythrin*.

CD1 molecules have been found to interact with the human γδ TCR. The first isolated human T cell clone showed CD1c autoreactivity (12) and other CD1c-autoreactive γδ TCR cells were found later (13, 14). Rare CD1d-restricted γδ TCR cells were identified by staining with CD1d tetramers loaded with α-Galactosylceramide (15) or with sulfatide (16, 17). Vδ1 was the Vδ chain used in both types of γδ cells and it was the predominant chain interacting with CD1d. The Vγ chain was found contacting α-Galactosylceramide (15), whereas in the case of sulfatide recognition, the Vγ chain interacted neither with CD1d nor with sulfatide (17).

Another surface molecule with a CD1d-resembling structure, which stimulates a rare population of γδ cells is the endothelial protein C receptor (EPCR) (18), a lipid-binding protein expressed by endothelial cells. One T cell clone expressing a Vδ5–Vγ4 TCR interacted with low affinity with EPCR, and this interaction was facilitated by CMV infection. The enhancing effect of CMV was not related to changes in EPCR lipid binding and remains poorly characterized.

Other MHC-like molecules interacting with mouse γδ cells are TL 27b (19), TL 10b (11, 20), and T22 (21). The structure of the specific TCR bound to the T22 molecule showed that germline-encoded residues of the CDR3δ loop were responsible for binding T22 in an orientation different from that seen in αβ TCR binding to MHC–peptide complexes (5). Human Vδ1-expressing cells were also found to interact with the MICA molecule (22). The Vδ1 TCR of these cells and the NKG2D, another MICA receptor, bound MICA in a mutually exclusive manner. The analysis of the binding kinetics suggested a model in which the initial contact between the γδ cells and the target cell is established by fast binding of MICA to NKG2D, followed by a much-prolonged interaction with the TCR.

Two other proteins were reported to stimulate human Vγ9Vδ2 cells, namely “an entity related to the mitochondrial F1 ATPase” expressed by some tumor cells and a delipidated form of apolipoprotein A-I (Apo A-I) (23). A soluble Vγ9Vδ2 TCR was found to interact with Apo A-I and bovine F1 ATPase in the low micromolar range by surface resonance (SPR) studies. When different Vγ9Vδ2 clones were compared, only some were positively influenced by the presence of Apo A-I, indicating a redundant role of this molecule.

A last set of proteins that were reported to stimulate human γδ cells are heat-shock proteins (24–26). Surface expression of these molecules was associated with targeting of γδ cells on different tumor cells (27).

All these findings suggest that antigen recognition by γδ T cells is different from that of αβ T cells. The different length of both Vγ and Vδ CDR3 loops also supports this conclusion. These loops are often critical for antigen binding in Ig and significantly contribute to peptide binding in αβ TCRs. When the CDR3 regions of Ig H and L chains are compared with those of TCR α, β, γ, and δ chains, the one from Ig H and TCR δ are the most variable in size and are significantly longer than Ig L and TCR γ chains, respectively (28). In contrast, TCR α and β pairing occurs with chains of nearly identical average CDR3 lengths. These important TCR structural differences have been related to a type of antigen recognition by γδ TCR similar to that of Ig (29).

## Small Molecules Stimulating γδ TCR Cells

Several studies have identified both human and mouse γδ T cells that were activated by small peptides, carbohydrates, and haptens. Initial studies showed the existence of human and mouse γδ cells recognizing MHC–peptide complexes expressed on the surface of antigen-presenting cells (APC) (10, 30–32). In contrast with human alloreactive αβ TCR cells, the number of alloreactive γδ cells remains quite rare. H2-peptide-specific γδ TCR cells were found in mice, after inhalation of ovalbumin (33). Whether these γδ cells recognize intact ovalbumin or its peptides was not investigated.

Mouse γδ TCR hybridomas recognizing peptides from heat-shock proteins were also described. Interestingly, these peptides were presented by non-MHC molecules and their recognition was sensitive to amino-acid changes in the peptide sequences and to the type of Vγ chain expressed by specific hybridomas (34). Another study described mouse γδ cells recognizing glycosylated peptides in which MHC class I-bound peptides with two or three sugars were stimulatory (35). The central position of the sugar in the peptide sequence was critical for stimulation (36), probably because of direct interaction with the γδ TCR.

Several studies have shown that in some patients with myopathies, γδ cells infiltrate the affected area and are associated with acute pathology (37). In one study, the infiltrating T cells were cloned and found to be oligoclonal (38). The TCR of these cells was composed of a Vγ1.3–Jγ1–Cγ1/Vδ2–Jδ3 heterodimer, which is not frequent in normal donors. This TCR recognized histidyl-tRNA synthetase, an antigen also recognized by anti-Jo-1 autoantibodies (39). The γδ TCR target epitope was strictly conformational, independent of post-translational modification, exposed on the surface of the intact protein, and mutagenesis studies showed that a short alpha-helical loop constituted part of the γδ stimulating epitope.

Recent studies reported the identification of two mycobacterial proteins, namely 1-deoxy-d-xylulose 5-phosphate synthase 2 and Rv2272 protein, which activated γδ T cells isolated from patients with pulmonary tuberculosis (40). Two peptides, 12 amino-acid long from each of these proteins, retained the capacity of stimulating a major population of γδ cells expressing a unique CDR3δ segment. Whether this recognition occurred by cognate interaction of the TCR with the peptides or via a dedicated presenting molecule was not investigated.

More recently, mouse γδ cells specific for small haptens have been identified (41). These T cells recognized cyanine 3, a synthetic fluorescent molecule, and 4-hydroxy-3-nitrophenylacetyl, a classical hapten. Another small molecule stimulating both murine and human γδ TCR cells is the algae protein phycoerythrin (PE) (42). The TCR of isolated cells directly interacted with the whole PE, thus indicating a B cell-like antigen recognition capability.

**Figure 1:**
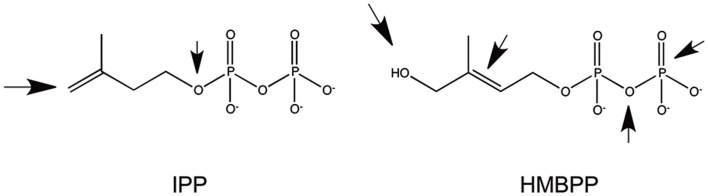
**Structure of the two most common phosphorylated metabolites stimulating Vγ9Vδ2 cells**. The arrows indicate the parts of the molecules whose modifications significantly reduced immunogenicity.

Another type of small antigens stimulating γδ cells is represented by phosphorylated metabolites generated in the mevalonate pathway in eukaryotic cells or in the methyl erythritol pathway in bacteria and in some eukaryotes. The most representative stimulatory molecules are isopentenyl pyrophosphate (IPP) (43, 44) and (E)-4-hydroxy-3-methyl-but-2-enyl pyrophosphate (HMBPP) (45) (Figure 1). Both are very small molecules with a molecular weight of 245 and 262, respectively, and are composed of one isoprene unit to which a diphoshate is attached. Small changes in the structure of these ligands, either on the phosphate or the isoprene moieties, profoundly affect the γδ cell stimulatory capacity. Several analogs of these compounds have been synthesized with an intermediate stimulatory capacity between that of IPP and of HMBPP [reviewed in Ref. (46)]. The same population of γδ cells also recognizes some tumor cells. This recognition is ascribed to the abnormally elevated production of IPP by tumor cells, as result of changes in the regulation of their mevalonate metabolic pathway (47). All of these findings show that γδ cells may cross-react to phosphorylated metabolites accumulating inside tumor cells and to metabolites released by bacterial cells in the microenvironment. Importantly, when bacteria infect target cells, they induce alteration of the host mevalonate pathway by subverting several regulatory mechanisms (48). These alterations lead to a transient and acute accumulation of IPP, which is then responsible for the activation of γδ cells. In conclusion, γδ cells may recognize (i) tumor cells that accumulate IPP; (ii) bacterial metabolites such as HMBPP, and (iii) cells accumulating IPP following infection with bacteria not producing HMBPP.

An important finding is that these metabolites stimulate the human cells expressing the Vγ9Vδ2 heterodimer and also other primate γδ cells, which express TCR heterodimers closely resembling the Vγ9Vδ2 TCR (49). The role of the CDR3 sequences of Vγ9Vδ2 TCR in antigen recognition remains debated. Sequence analysis of T cell clones reacting to IPP and HMBPP (50–54) showed CDR3 regions of different length and sequences, thus indicating that they are unlikely to be involved in direct contact with such small antigens. In addition, most peripheral, but not thymic Vγ9Vδ2, clones contained Val or Leu or Ile amino acids at position 97 of the CDR3δ (55), raising the possibility of a selection for hydrophobic residues during expansion in peripheral blood. The conclusions of these investigations were in line with more recent data in which the mutation of the TCR Vγ9 and Vδ2 genes suggested that a large TCR footprint is involved in antigen stimulation (56), probably because the TCR interacts with molecules much larger than the small stimulatory metabolites.

Although the stimulation of γδ cells by phosphoantigens has been described for many years, important cell biology aspects of how the Vγ9Vδ2 TCR engages these molecules remained poorly defined. Classical studies of antigen presentation were performed in different laboratories and provided useful information to the understanding of how this interaction may occur. APC were necessary for this activation and only human APC could activate Vγ9Vδ2 cells, suggesting that antigen-presenting molecules or surface-expressed co-stimulatory molecules were necessary (57). These observations were confirmed in other studies (58, 59). Cells from many different human tissues (47), from different donors, and APC neither expressing MHC class II molecules nor β2-microglobulin (57) were also stimulatory indicating that the required presenting/accessory molecule is ubiquitous, non-polymorphic, and species-specific.

Fixed APC maintained the capacity to stimulate Vγ9Vδ2 cells as shown by lymphoma Daudi cells (25), which accumulate endogenous IPP (47). Fixed APC remained stimulatory also when exogenous IPP was added after fixation (60), indicating that metabolically inactive APC remain stimulatory. This information also suggested that the exogenous antigen does not require specific internalization into APC and that the presenting molecule is already expressed in a stimulatory form on Daudi cells before fixation and so it remains after fixation. In one study, the responsiveness of Vγ9Vδ2 cells to a crude mycobacterial lysate was investigated (61). When monocytes were first pulsed and then fixed, they retained the stimulatory capacity, whereas they were not stimulatory if pulsed after fixation. This study also showed that chloroquine increased the antigen-presenting capacity of APC and this effect was not associated with inhibition of lysosome acidification. It was suggested that chloroquine facilitates antigen presentation by decreasing the degradation or turnover of surface presenting molecules. These studies underlined the importance of surface molecules that can be fixed in a stimulatory state upon antigen binding.

Several laboratories also reported that phosphoantigens cannot be pulsed on APC, i.e., when APC are incubated with phosphoantigens, they immediately lose their stimulatory capacity upon washing (58, 60), probably caused by a weak binding to, and a fast dissociation from, the presenting molecule. This behavior is incompatible with a cytoplasmic stimulatory activity of phosphoantigens, and is instead in agreement with an extracellular role of a presenting molecule on APC. Consistent with a weak extracellular antigen binding, association of prenyl pyrophosphate antigens with the surface of APCs was reported only in the presence of 50- to 1000-fold higher concentrations of antigen than those required to stimulate γδ cells (62).

Another important finding was that the addition of the antigen to APC is immediately followed by T cell response. This was observed in several kinetic studies (58, 63, 64). Using cytosensor microphysiometry, γδ T cell activation was detected in <9 s after antigen addition (63). As charged compounds passively pass the plasma membrane with great difficulty (65) and require an active endocytic process, it is unlikely that phosphoantigens added to the extracellular milieu may accumulate in the cytoplasm of APC in <9 s. Such a fast T cell response is in agreement with the possibility that the antigen binds to surface molecules almost immediately and that internalization into the APC is not necessary.

Other clues to the nature of antigen binding comes from studies of stimulatory and inhibitory capacity of IPP (66) and HMBPP (63) analogs. Antagonistic activity was found with some ligands, and in one case, γδ cells became unresponsive after a very brief interaction (<5 min) with the antagonist compounds (66). This state of unresponsiveness was fully reversible but lasted at least 3 days even after removal of the antagonist, thus indicating that the interaction with the antagonist induced a significant change in γδ cells. This observation is in agreement with a model of antigen recognition in which antigenic molecules exposed on the cell surface bind phosphorylated metabolites and, according to their structure, an agonist or antagonist signal is induced in the interacting γδ cells. It remains intriguing that when competition studies were performed using methylene diphosphonates, higher doses of competitors were required to inhibit stimulation with IPP than with BrHPP (63) despite the fact that IPP is a weaker agonist than BRHPP (67). Further investigations are required to explain this unexpected result.

A significant finding was that the Vγ9Vδ2 TCR enters the immune synapse in the absence of antigen (68), thus suggesting that this TCR may interact with molecules already exposed on the APC surface in the absence of phosphoantigens. This interaction is enough to form an APC-T cell synapse, although it is not capable of inducing a full γδ cell response, which instead requires the presence of the antigen.

## The Role of Butyrophilins in Stimulating γδ Cells

**Figure 2:**
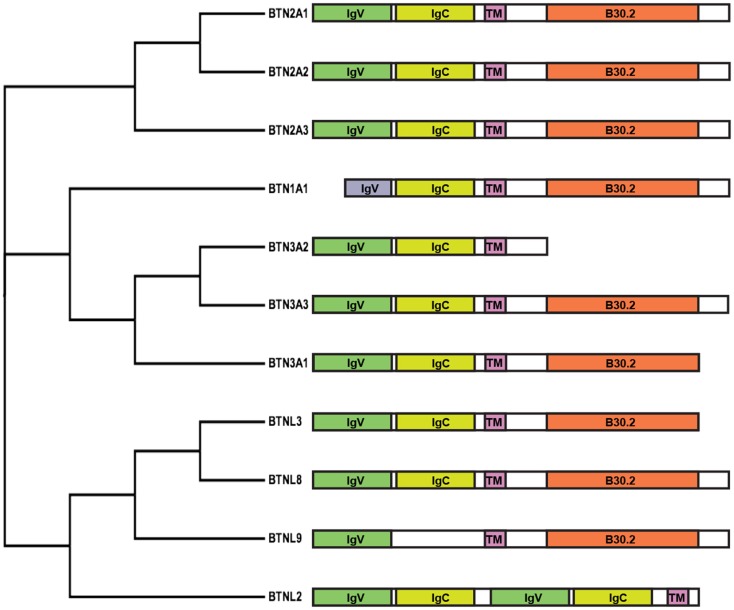
**Cladogram and schematic diagram of human BTN and BTNL family members**. Protein sequences were aligned with ClustalW2 and the tree rendered using FigTree software (tree.bio.ed.ac.uk/software/figtree). Diagram shows the domain organization of members belonging to the BTN family consisting of two extracellular Ig-like domains, IgV and IgC, a single-pass transmembrane domain (TM) and an intracellular B30.2 domain. Exceptions include BTN1A1, consisting of a short IgV-like domain; BTN3A2, lacking a B30.2 domain; BTNL9, containing a single Ig-like domain; and BTNL2, consisting of four extracellular Ig-like domains and lacking a B30.2 domain.

An important advancement was made by the identification of Butyrophilin (BTN) 3A1 (BTN3A1) as the molecule required to stimulate Vγ9Vδ2 cells by phosphoantigens (69, 70). This molecule belongs to the family of BTN (Figure 2), which have been attributed a series of functions including immunomodulation (71, 72) and induction of maturation of mouse thymic Vγ5Vδ1 cells (73). BTN proteins can be involved in milk fat globule formation in the lactating mammary tissue of cows (74), or in dampening and inhibiting immune reactivity (71, 72).

The BTN3 family is conserved together with the TCR Vγ9 and Vδ2 genes in higher primates and in some species of placental mammals, but not in rodents (75). The common occurrence or loss of these three genes suggested their co-evolution based on a functional relationship.

Butyrophilins are structurally very similar to molecules of the B7 family. A major characteristic shared among the B7, BTN, and butyrophilin-like (BTNL) families is the two extracellular immunoglobulin-like domains – one IgV-like and one IgC-like domain. Some members, like BTNL2 and B7 homolog 3 (B7H3), instead comprise four extracellular immunoglobulin-like domains. Like several members of the B7 family (76), BTN proteins have also been found to form dimers (77), although their occurrence on the surface of APC remains to be confirmed. Structural studies with soluble BTN3 molecules have shown the formation in solution of two types of BTN3 dimers (77). One type is formed by juxtaposition of the C-like domains (head-to-head dimer), resembling the dimers formed by B7 family members. In contrast, the second type of dimer was characterized by an asymmetric head-to-tail binding. Fluorescence resonance energy transfer experiments suggested that the head-to-head dimers are more frequently formed, at least in solution.

Importantly, when reconstitution experiments were performed by transfecting the BTN3A1 gene in mouse cells, it was found that this protein alone is not sufficient to restore the stimulation of Vγ9Vδ2 cells (69, 70, 78), suggesting that additional molecules are necessary.

A common feature of the BTN and BTNL families (with the exception of BTN3A2 and BTNL2) is the presence of intracellular domains with a structure resembling the B30.2 domain found in more than 150 other human proteins (79). This domain forms protein–protein interactions with various cytoplasmic molecules with different activities (80).

One study describing the stimulatory role of an anti-BTN3A1 monoclonal antibody (mAb) (69) provided early insights into the mechanism of BTN3A1–phosphoantigen presentation. When this antibody was added to target cells expressing BTN3A1, it induced the activation of Vγ9Vδ2 cells independently of the presence of phosphoantigens. A second anti-BTN3A1 mAb was instead inhibitory. These findings raised a series of new questions associated with the occurrence of both stimulatory and inhibitory anti-BTN mAbs. This issue was further studied by resolving the structure of BTN3A in association with the two mAbs (77). Indeed, while the inhibitory antibody bound to the distal part of the V-like domain, the stimulatory antibody bound to a more membrane proximal region of the V-like domain. The activatory antibody was also found to be compatible with *in vitro* formation of BTN3 homodimers in which the C-like domains of two BTN3 molecules interact with each other, as reported for other B7-like molecules. The authors speculated that the capacity of this antibody to facilitate this type of dimers was associated with the stimulatory capacity of this mAb, whereas the inhibitory mAb prevented BTN3 homodimerization.

A second study used a genetic approach to identify the chromosomal loci encoding the gene required for stimulation of Vγ9Vδ2 cells (70). By using a panel of mouse–human somatic cell hybrids, the telomeric region of human chromosome 6 was identified as important. By using a second series of somatic hybrids with truncations in this region, a closer genetic mapping identified 14 candidate genes, and among those BTN3A1 was found necessary for stimulating γδ cells. Transfection and knock out studies confirmed that while BTN3A1 was important, BTN3A2 and BTN3A3 had no apparent role in stimulating Vγ9Vδ2 cells. Additional experiments investigated the mechanism of BTN3A1 stimulation. A recombinant BTN3A1 protein containing only the V-like domain showed binding to IPP and HMBPP. This was investigated using three different approaches, namely SPR, mass spectrometry of intact BTN3A1–antigen complex, and structural analysis of BTN3A1–IPP and HMBPP complexes. These studies showed a weak interaction of the two phosphoantigens with BTN3A1 and indicated their mode of binding. Additional studies addressed the important issue of whether the Vγ9Vδ2 TCR makes cognate interaction with the BTN3A1–phosphoantigen complexes. This aspect was initially investigated by SPR and then by surface-enhanced Raman scattering (SERS), a technique capable of detecting very weak protein–protein interactions. These studies revealed that only a soluble Vγ9Vδ2 TCR interacted with the complex, and neither soluble Vγ9Vδ1 TCR nor αβ TCR used as controls. The Vγ9Vδ2 TCR weakly interacted with the recombinant BTN3A1 in the absence of phosphoantigens and this interaction was enhanced by addition of IPP (70).

**Figure 3:**
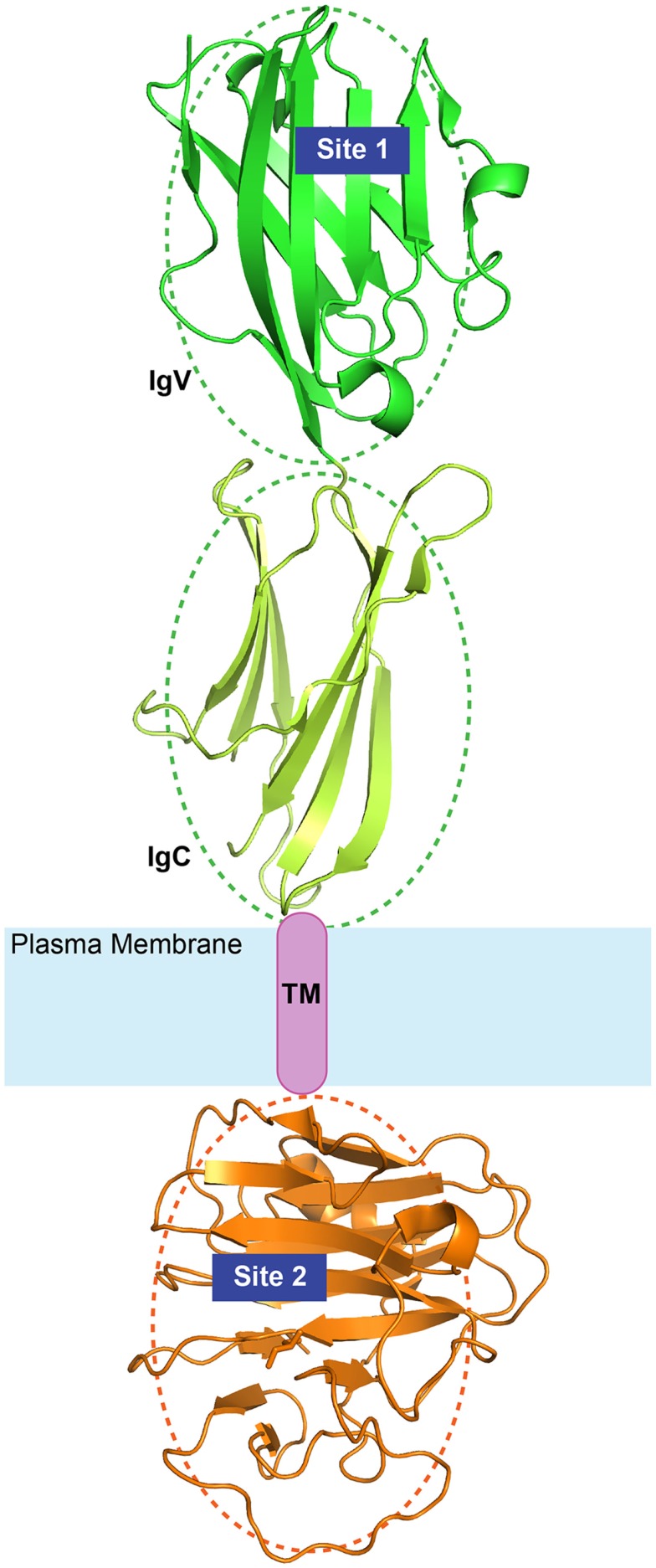
**Diagram of BTN3A1 topology**. The extracellular Ig-like domains (green) and the intracellular B30.2 domains (orange) are illustrated here with available crystal structures (PDB IDs: 4F80 and 4N7U). The relative orientation of the domains is arbitrary as depicted by the dotted ovals. Site 1 and Site 2 represent the two identified binding sites of phosphoantigens.

Another important finding was that when the cytoplasmic B30.2 domain of BTN3A1 was grafted on the non-stimulatory BTN3A3 molecule, stimulation of Vγ9Vδ2 was restored (69). Thus, both the extracellular and the cytoplasmic domains of BTN3A1 were required (Figure 3). The importance of intracellular domains has been already reported in the field of antigen presentation. Indeed, the cytoplasmic domains of other antigen-presenting molecules, for example, CD1 molecules, are involved in proper internalization, endosomal recycling, and in the physiological presentation of lipid antigens (81). The cytoplasmic domains of several presenting molecules associate with different protein partners and each of these interactions contribute to antigen presentation and productive T cell activation.

In more recent studies, binding of IPP and HMBPP to the B30.2 domain and not to the V-like domain of BTN3A1 was reported (82, 83), and mutagenesis studies of the B30.2 domain of the non-stimulatory BTN3A3 where an amino-acid change in the putative antigen binding pocket to that of BTN3A1 conferred binding of HMBPP and γδ cell stimulatory capacity (82). In this latter study, no binding of the TCR to the V-like domain of BTN3A1 was detected and it was proposed that the B30.2 domain is important because it binds phosphoantigens and with unknown mechanisms it induces the activation of γδ cells. Although interesting, this hypothesis is inconsistent with the published literature discussed above. The incapacity of detecting phosphoantigen and TCR binding to the V-like domain of BTN3A1 might be ascribed to technical reasons, for example, utilization of techniques not capable of detecting weak protein–protein interactions and lack of adequate control of the proper conformation of recombinant molecules studied.

As described above, a large number of data obtained in several laboratories indicated that phosphoantigens must be present outside the APC to stimulate γδ cells and these data are not compatible with a model in which exogenous phosphoantigens must first be internalized into the cytoplasm to become active. The fact that the B30.2 domain of BTN3A1 binds the phosphoantigens *in vitro* is not proof that similar binding occurs *in vivo*. This is a main issue that has not been experimentally tested, but is fundamental to proposing the hypothesis of the intracellular mode of phosphoantigen activity. As there is common agreement that the B30.2 domain has a major role in activation of Vγ9Vδ2 cells, other possibilities should be considered to explain its mode of function. One hypothesis is that the B30.2 domain binds to one or several cytoplasmic proteins instrumental for the correct display of BTN3A1. A second possibility is that the B30.2 domain is necessary for the correct recycling of BTN3A1 to endosomal compartments. According to this latter possibility, the B30.2 domain would resemble the cytoplasmic tails of other antigen-presenting molecules that bind to cytoplasmic signaling partners and direct BTN3A1 trafficking to compartments where the antigen is loaded and unloaded. These alternative mechanisms of action require the presence of unique motifs in the B30.2 domain. It is possible that some of these important motifs could have been lost in the mutagenesis study (82).

**Figure 4:**
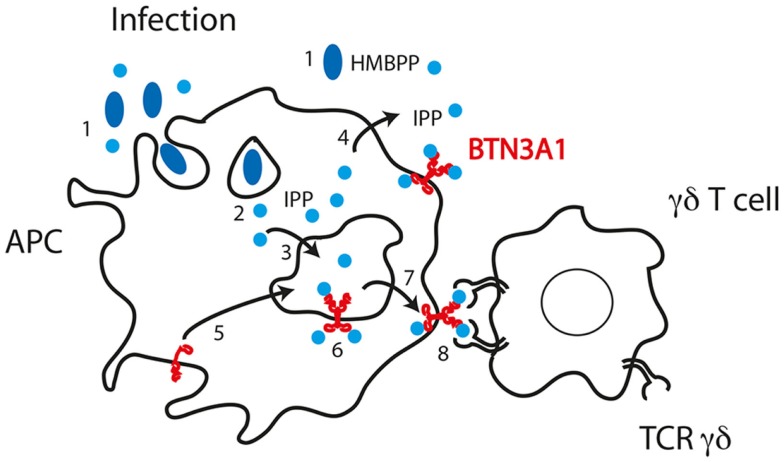
**Model of phosphoantigen presentation according to published data and mechanisms of phosphoantigen handling by APC**. (1) Bacteria release HMBPP in the microenvironment or are internalized in phagosomes where HMBPP may be stored. (2) HMBPP passes phagosomal membranes and reaches BTN3A1 with unclear mechanisms. (3) Cytoplasmic IPP accumulated in the cytoplasm passes the membrane of endosomal compartments. (4) IPP may also pass the plasma membrane. (5) BTN3A1 traffics from plasma membrane to endosomal compartments where it encounters IPP. (6) IPP may also interact with the B30.2 cytoplasmic domain of BTN3A1. (7) Dimers of BTN3A1 containing IPP are formed and traffic to the plasma membrane. (8) The TCR Vγ9Vδ2 interacts with the BTN3A1 dimers and IPP facilitates this interaction.

On the basis of all these data, we propose a model of activation of Vγ9Vδ2 cells by phosphoantigens whereby the Vγ9Vδ2 TCR makes cognate interaction with the BTN3A1 molecule (Figure 4). The interaction of the TCR with the V-like domain of BTN3A1 is positively influenced by the presence of phosphoantigens through two possible mechanisms. The first one takes into account that the phosphoantigens interact with the TCR, thus increasing the overall affinity of interaction as suggested by SERS experiments (70). Alternatively, phosphoantigen binding may induce a conformation change in BTN3A1, which in turn interacts with the TCR, leading to a full activation of γδ cells. Both these possibilities are in line with the inhibitory function of antagonist analogs (63, 66). The presence of an extracellular V-like domain is not sufficient to activate Vγ9Vδ2 cells, as shown by the relevance of the B30.2 cytoplasmic domain (69). This domain could also bind phosphoantigens (82), and it remains difficult to envisage how γδ cell activation is induced upon this interaction. One hypothesis is that upon phosphoantigen binding to the B30.2 domain, the extracellular domains of BTN3A1 assume a new conformation, which promotes stable contact with the γδ TCR. Alternatively, phosphoantigen binding to the B30.2 domain could facilitate proper trafficking and membrane localization of BTN3A1. It is important to underline that binding to the V-like and B30.2 domains may not be necessarily mutually exclusive. Phosphoantigen binding to both domains may be needed for appropriate γδ cell stimulation. A new series of mutagenesis and reconstitution experiments in BTN3-deficient APC are required to properly address this issue.

An important aspect is how endogenous ligands, such as IPP that is synthesized in the cytosol, are loaded onto the extracellular V-like domain of BTN3A1. Such an event implies that IPP crosses an endosomal membrane or the plasma membrane. Data in our laboratory indicate that a dedicated transporter is required for presentation of endogenous IPP. Remarkably, the transporter is required only when IPP accumulates within the APC and not when it is provided exogenously.

Although great advancement has been achieved in understanding the mechanisms of Vγ9Vδ2 cell activation and phosphoantigen presentation, many questions remain. For example, how is the surface expression of BTN molecules regulated, in which cellular compartment do they traffic, do they associate with other molecules intracellularly and/or on the plasma membrane, and how do phosphoantigen antagonists inhibit BTN3A1? As members of the BTN3 family have been found to interact with other surface proteins, which are not γδ TCR and are not broadly expressed (84), it will be also important to identify these other partners and investigate their role in T cell responses.

The light shed by BTN3A1 on the conundrum of antigen presentation to Vγ9Vδ2 cells has exposed only the tip of an iceberg. It will be very interesting to follow the evolution of this field and the implications of BTN in stimulating other types of γδ cells.

## Conflict of Interest Statement

The authors declare that the research was conducted in the absence of any commercial or financial relationships that could be construed as a potential conflict of interest.
